# Super-Resolution Microscopy as a Versatile Tool in Probing Molecular Assembly

**DOI:** 10.3390/ijms252111497

**Published:** 2024-10-26

**Authors:** Nan Sun, Shiwei Bai, Luru Dai, Yi Jia

**Affiliations:** 1 National Engineering Research Center for Colloidal Materials, Shandong University, Jinan 250100, China; sunnan@sdu.edu.cn; 2 Beijing National Laboratory for Molecular Sciences (BNLMS), CAS Key Lab of Colloid Interface and Chemical Thermodynamics, Institute of Chemistry Chinese Academy of Sciences, Beijing 100190, China; baisw0101@163.com; 3 School of Chemical Science, University of Chinese Academy of Sciences, Beijing 100049, China; 4 Wenzhou Key Laboratory of Biomedical Imaging, Wenzhou Institute, University of Chinese Academy of Sciences, Wenzhou 325000, China; dai@ucas.ac.cn

**Keywords:** super-resolution microscopy, molecular assembly, interactions, dynamics

## Abstract

Molecular assembly is promising in the construction of advanced materials, obtaining structures with specific functions. In-depth investigation of the relationships between the formation, dynamics, structure, and functionality of the specific molecular assemblies is one of the greatest challenges in nanotechnology and chemistry, which is essential in the rational design and development of functional materials for a variety of applications. Super-resolution microscopy (SRM) has been used as a versatile tool for investigating and elucidating the structures of individual molecular assemblies with its nanometric resolution, multicolor ability, and minimal invasiveness, which are also complementary to conventional optical or electronic techniques that provide the direct observation. In this review, we will provide an overview of the representative studies that utilize SRM to probe molecular assemblies, mainly focusing on the imaging of biomolecular assemblies (lipid-based, peptide-based, protein-based, and DNA-based), organic–inorganic hybrid assemblies, and polymer assemblies. This review will provide guidelines for the evaluation of the dynamics of molecular assemblies, assembly and disassembly processes with distinct dynamic behaviors, and multicomponent assembly through the application of these advanced imaging techniques. We believe that this review will inspire new ideas and propel the development of structural analyses of molecular assemblies to promote the exploitation of new-generation functional materials.

## 1. Introduction

Molecular assembly can organize individual molecules to form intricate structures and specific functional systems [1,2,3,4,5]. Essential structures in living organisms (cell membranes, organelles, chromatin, and cytoskeletons) are formed through molecular assemblies and vital cellular activities (RNA transcription, DNA replication, protein translation, membrane remodeling) that all involve the molecular assembly process. Inspired by nature, artificial molecular assembly beyond individual molecules has been created to fabricate novel materials [6,7,8]. Both natural and artificial molecular assemblies formed by non-covalent interactions provide unique structures, which have a variety of sizes and morphologies that cannot be constructed by synthetic chemistry based on covalent bond linkage [9,10]. The strategy of molecular assembly has had great achievements, enabling the construction of diverse functional materials that have a wide range of applications in various fields.

Investigating the structures and functions of molecular assemblies thoroughly is vital for the development of assembled materials with various functions [7,11]. Comprehending the mechanism is even essential for complex molecular assembly systems, as their composition, dimensions, dynamics, and hierarchical structure pose a great challenge for the techniques of characterization [11]. Various techniques have been carried out to detect the analyses of molecular assemblies, including crystallography (small-angle X-ray/neutron scattering, X-ray diffraction), spectroscopy (UV-visible, fluorescence, circular dichroism (CD), and NMR), microscopy (transmission electron microscopy (TEM), atomic force microscopy (AFM), confocal laser scanning microscopy (CLSM), and scanning electron microscopy (SEM)), and so forth [12]. Each technique has its limitations and advantages related to the contrast and operational requirements, therefore, using multiple complementary techniques is usually recommended. For instance, crystallography and spectroscopy techniques possess the ability of analyzing the information on the molecular structure and interaction and the information on the microstructure of the materials, further predicting their macroscopic properties; however, without the directly observed image information [13]. SEM, TEM, and AFM offer label-free detection and high spatial resolution, however, without enabling non-invasive and multicolor imaging, because of the limited sample permeation [13,14]. FM can be used to detect the native conditions of materials with multicolor imaging, but with a very limited resolution [15,16]. CLSM can be used as powerful tool to visualize multiple fluorescent signals from materials with also limited spatial resolution about 200 nm, which is not suitable for specimens with smaller size [17].

Super-resolution microscopy (SRM) techniques have greatly revolutionized the way that we study biological systems since their advent, breaking the diffraction limit and allowing the observation of samples as low as tens of nanometers and even at the single molecule level. Since the acknowledgement of the Nobel Prize in Chemistry in 2014 [18], SRM techniques have gained enormous interest from the fields of biology to other realms of science, including biomedicine, physics, plant science, and food science [19,20,21,22,23,24,25,26]. In recent years, SRM technologies have also become increasingly influential in materials science and chemistry as a method to reveal the dynamics and structures of assembled materials, which facilitate the visualization of materials with minimal invasiveness in operando [22,27]. More importantly, the use of SRM has also revolutionized the way that we comprehend supramolecular chemistry and molecular assemblies, complementing existing techniques and allowing us to address the individual dynamics and structures of molecular assembly by visualizing the individual self-assembled objective with its unprecedented temporal and spatial resolutions [17,28]. Different from TEM and SEM, SRM enables multicolor imaging with appropriate fluorescent labelling for detecting molecule interactions and provide in situ imaging, which can avoid the invasion of sample preparation and the drying or freezing procedure [17]. SRM herein has played an important role in the investigation of molecular assemblies and promoted the research of the structure of complex assembled materials [28,29,30].

**Figure 1:**
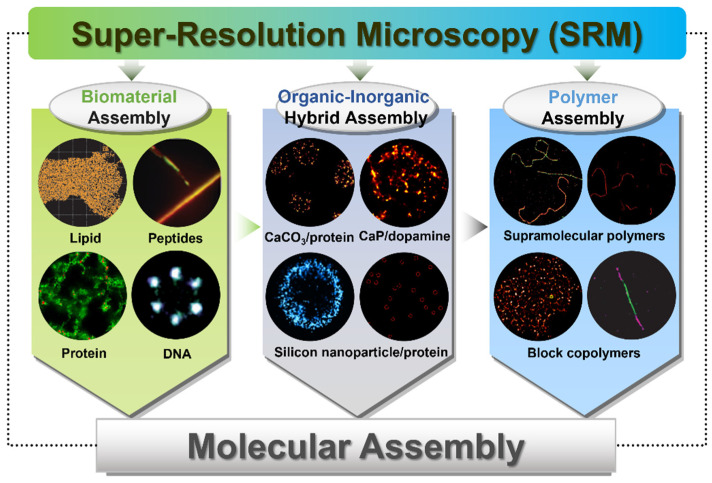
Schematic illustration of SRM application in molecular assembly research.

In this review, we summarize the representative reports and highlight the advancements in the application of SRM in the research of molecular assembly (Figure 1), from the biomolecule assemblies (lipid-based, peptide-based, protein-based, and DNA-based), to organic–inorganic hybrid assemblies and even polymer assemblies. All these cutting-edge techniques help scientists to obtain deeper understandings of the dynamic structures, assembly process, functions, and dynamics of the assembled architectures and the relationships to their performances. This review provides guidelines for the evaluation of the dynamics of molecular assemblies, assembly and disassembly processes with distinct dynamic behaviors, and multicomponent assembly through the application of these advanced imaging techniques. Finally, we discuss the upcoming improvement directions and trends in the future. We believe that this review will inspire new ideas, propel the development of structural analyses of molecular assemblies, and promote the exploitation of the next-generation functional materials.

## 2. Classification of Super-Resolution Imaging Methods and Working Mechanisms

Generally speaking, super-resolution optical imaging technology is mainly divided into two categories: the first type is hidden wave detection imaging, which can detect the information on the surface of the object only, reaching a resolution of 20–50 nm [31,32,33]; the other category is high-resolution far-field optical microscopy, which can be spanided into three groups, as detailed in Table 1. Based on the different imaging mechanisms, structured illumination microscopy (SIM) [34,35], stimulated emission depletion (STED) [1,17,36], and single-molecule/-particle localization (SMLM) [37,38,39,40] are usually discussed. Specifically, among them, SMLM can reach a resolution of about 20 nm, the laser power required is lower, and the optical path is relatively more simple and easier to realize, which has been applied in many laboratories. Here, we emphasize several commonly used SRM techniques and their mechanisms.

SIM is one of the most widely used optical super-resolution techniques, which is excited by a specific spatially structured pattern of light that produces interference patterns. Using SIM, a super-resolution image with a lateral resolution of approximately 120 nm and an axial resolution of 350 nm can be achieved [34,35]. Samples prepared for standard fluorescence microscopy can be investigated using SIM, without requiring significant additional sample preparation effort, which makes it an appealing imaging technique [35].

In STED microscopy, the high resolution is achieved using stimulated emission to reduce the effective fluorescence emission area [58]. Two beams of illumination are required for the typical STED microscopy system, one for excitation and the other for depletion. When the fluorescence molecules within the range of the diffraction spot are excited by laser irradiation, the electron will convert to the excited state. The depletion light causes some electrons in the periphery of the excited light spot to return to the ground state by stimulated emission, while the rest of the excited electrons in the center of the excited light spot continue to return to the ground state by spontaneous fluorescence without being affected by the depletion light. Since the wavelength and propagation direction of the fluorescence emitted by the stimulated emission and the self-fluorescence are different, the photons received by the detector are generated by the fluorescence sample located in the center of the excitation spot through the self-fluorescence mode. Therefore, the luminous area of fluorescence can be reduced effectively, resulting in the improvement of the resolution of the system, with an axial resolution of ~150 nm and a lateral resolution of ~25–80 nm [58,59]. Recently, tremendous efforts have been devoted to the investigation of fluorophores for the STED technique and these materials include inorganic or organic luminescent materials, fluorescent proteins or nanoparticles, and aggregation-induced emission (AIE) luminogens [58].

SIM and STED are designed to image multiple fluorophore ensembles, differently, SMLM possess the ability to detect individual fluorophores with the optimum spatial resolution down to 5 nm [60]. Based on their imaging mechanisms, SMLM techniques usually require other photoswitching or photoactivatable probes. While the single fluorescent molecular is in the on-state, the fluorescent can be localized; while in the non-fluorescent off-state, it cannot be localized [61]. By analyzing and reconstructing all the obtained images, the super resolution image can be built point by point. Typically, SMLM techniques are divided into three categories based on the manner of the probe’s transition between the active and inactive states, including photoactivation approaches (PALM, fPALM), photoswitching methods (STORM, dSTORM), as well as reversible binding techniques (PAINT, DNA-PAINT, and BALM) [38].

## 3. Molecular Assemblies Investigated by Super-Resolution Microscopy

### 3.1. Biomolecular Assemblies

SRM has widely used in the study of biomaterial assemblies [27,62]. Different from SEM and TEM, SRMs are equipped with the ability of offering excellent spatial resolution with appropriate fluorescent labelling, enabling the detection of the molecule interaction with multicolor imaging [17]. In this section, we will summarize the advanced examples on the exploration of SRM in the field of biomaterial assembly, including lipids, peptides, proteins and DNA. By using of these cutting-edge techniques, scientists are able to look at the structures, functions and dynamics of novel molecular architectures, deduce the relations between the structures and the optimal performances, finally accelerating the development of the new-generation functional materials. Due to the respective benefits and constraints of each method, the combination of multiple complementary techniques is highly recommended by taking into account the specific operational needs and contrasts [63,64,65].

#### 3.1.1. Lipid-Based Molecular Assemblies

Due to their highly tunable formulations and distinctive advantages, liposomes and lipid nanoparticles (LNPs) are regarded as the leading options for biomimetic drug delivery platforms [66]. A mechanistic understanding of the assembly dynamics and mechanism, and recapitulating the interactions between the biological membrane and nanoparticles, are essential for designing and constructing cell-targeted carriers for precision medicine [66,67]. With a size range of 50~200 nm, the typical lipid assembly can be perfectly visualized using SRM. With the inherent hydrophobic nature of the bilayer interior, the liposomes are ideally suitable for PAINT using lipophilic probes.

**Figure 2:**
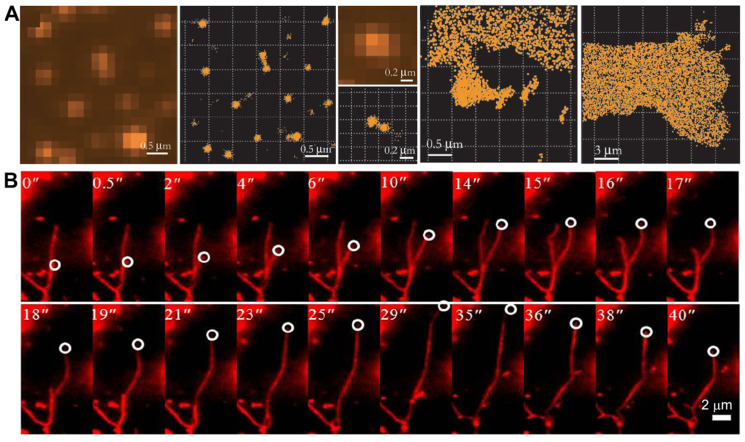
(**A**) Imaging of vesicles: images of a supported bilayer on glass and high-resolution synthetic image of the contour of a supported bilayer. Reproduced with permission from [53]. (**B**) Dynamic characteristics of OO4 lipid assembly. Reproduced with permission from [71].

Hochstrasser et al. reported the first example of using PAINT to observe 100 nm unilamellar vesicles, as well as a supported lipid bilayer, using the hydrophobic probe Nile Red (Figure 2A) [53]. With its fluorescence emission strong in apolar environments but almost negligible in water, Nile Red was demonstrated to be an ideal probe in PAINT imaging. The association time of Nile Red to lipids is about 6 ms, which proved to be suitable for on/off switching and enabling single-molecule localization [53]. Lately, Hochstrasser’s group also reported the sub-diffraction optical imaging of lipid-phase separated regions with a nanometer resolution [68]. They combined a fluorescent probe of Merocyanine 540 that is sensitive to the lipid phase with PAINT to distinguish the gel- and liquid-phase nanoscale domains of the lipid bilayers supported on glass [68]. The population difference of single-molecule fluorescence could burst in the different phase regions because of the monomer–dimer equilibrium of MC540 in the membranes. The lateral phase separation of distinct lipids can be visualized using PAINT, which was proven to result in the formation of small domains within membranes. This method can also be extended to other binary or ternary lipid models or natural systems, providing a promising new super-resolution strategy.

Moreover, the development of spectrally resolved PAINT or sPAINT has enabled the concurrent recording of the spatial location and emission spectrum of individual dye molecules to a super-resolve image [69,70]. sPAINT can generate information-enriched md-SR images through the use of spectrally responsive fluorophores of the phenoxazone-based dye Nile Red [69]. Using sPAINT, the researchers super-resolve exploited the biological structures in the hydrophobicity domain of Nile Red, which was sensitive to the hydrophobicity of its environment [69]. Further study of synthetic lipid vesicles with a known composition has also validated this point. The hydrophobicity of amyloid aggregates implicated in neurodegenerative diseases has also been revealed using super-resolve sPAINT, as well as the hydrophobic changes in the membranes of mammalian cells. This technique could be readily integrated by placing a transmission diffraction grating within the optical pathway of a localization-based super-resolution microscopy (SRM) system. This setup allows for the concurrent extraction of all the relevant information from a single image plane.

Beyond the assessment of size and shape, gaining knowledge about the internal architecture of micelles and vesicles, as well as the dynamic interactions of particular liposome systems with cellular structures, will also provide valuable information [71,72,73]. Ulrike Alexiev and co-authors investigated the morphology of lipid nanocarriers using single-molecule fluorescence microscopy, directly visualizing the distribution of drugs within the nanostructured lipid carriers (NLCs) on the nanometer scale [72]. This approach will aid in tracking the precise distribution of drugs inside the NLCs, revealing the presence of two types of drug-loaded nano-compartments of varying sizes, which occupy up to approximately 50% of the volume of NLCs.

Attentions devoted to the research of lipid nanoparticles and the modification of their surfaces have been regarded as one of the major mechanisms of nanoparticle-cell interaction [66,67]. By applying the single-molecule imaging method, acting as a reliable and accurate quantificational tool, Van Oijen and co-workers determined the ligand density and stoichiometry on the surface of liposomes [73]. This method allowed for the comprehensive characterization of novel ligand-directed liposomes and could ultimately facilitate the translation of these nanotherapies from laboratory to clinic applications. Li and co-authors reported a dynamic interaction involving a particular liposome system using SRM, which involves lysine-based amino-functionalized lipid (OO4) and NIH 3T3 cells (Figure 2B) [71]. The assembled liposome system, which migrated towards the nucleus by forming aggregates, dispersed into moving vesicles and tubules in the cytosol. These liposomes underwent rapid movement through a dynamic mechanism, traveling at a pace that was roughly five to ten times faster than the typical transport vesicles found in cells. More interestingly, the induced tubules exist in various states, such as extending, retracting, or fluctuating along the cytoskeleton, exhibiting highly dynamic behavior with rapid movement, disintegration, and frequent fusion. The discovery made using the dSTORM technique offers an innovative conceptual approach for studying cellular dynamics, which is expected to enhance the exploration of membrane-mediated transport processes.

Owing to the distinctive properties of liposomes, there still exist both strengths and weaknesses of SRM studies. In terms of practicality and ease of use, the hydrophobic nature of the lipid bilayer allows for PAINT imaging to be carried out with polar probes. Nevertheless, lipid-based structures pose challenges for other SRM techniques like STORM or STED, primarily because of their instability and the complexities associated with fixation. The new journey in liposome nanoscopy imaging will start along with the developing of novel sample labelling and preparation procedures.

#### 3.1.2. Peptide-Based Molecular Assemblies

On account of their tunable assembly pathways, programmable structures, and versatile functions, peptides have attracted increasing attention in supramolecular assembly [74]. Being widely used in multiple fields, peptide-based supramolecular materials have been proven to be one of the most important engineering materials [1,74,75,76]. Gaining insight into the structure and function of peptide-based assembly materials at the single-molecule level is of great significance. This understanding is essential for advancing the development of improved materials for engineering purposes [77,78,79,80,81,82]. SRM techniques help to provide powerful tools for uncovering the spatial arrangement and interactions of peptide molecules at the nanoscopic level [83,84,85,86,87,88,89].

Meijer et al. reported the dynamics of peptide amphiphile (PA) nanofibers by two-color STORM imaging. They addressed the distribution of molecules along the fibers during exchange, investigated the heterogeneity among supramolecular nanofibers, and proposed a mechanism involving the transfer of monomers and small clusters [83]. The presence of both kinetically active and dynamically inert regions within the aggregate structure further illustrated the structural variability in PA nanofibers, which suggests that this intriguing dynamic behavior might have a great influence on the biological performance of PA supramolecular systems. Li and co-authors elucidated the dynamic morphological changes of the enzyme-instructed peptide-based supramolecular assemblies within cancer cells with a resolution below 50 nm using dSTORM [84]. The morphology of the hydrogel formed using peptide-based supramolecular assemblies was also visualized in vitro using dSTORM, revealing a thin nanofiber with a full width at half maximum (FWHM) of 47.5 nm.

**Figure 3:**
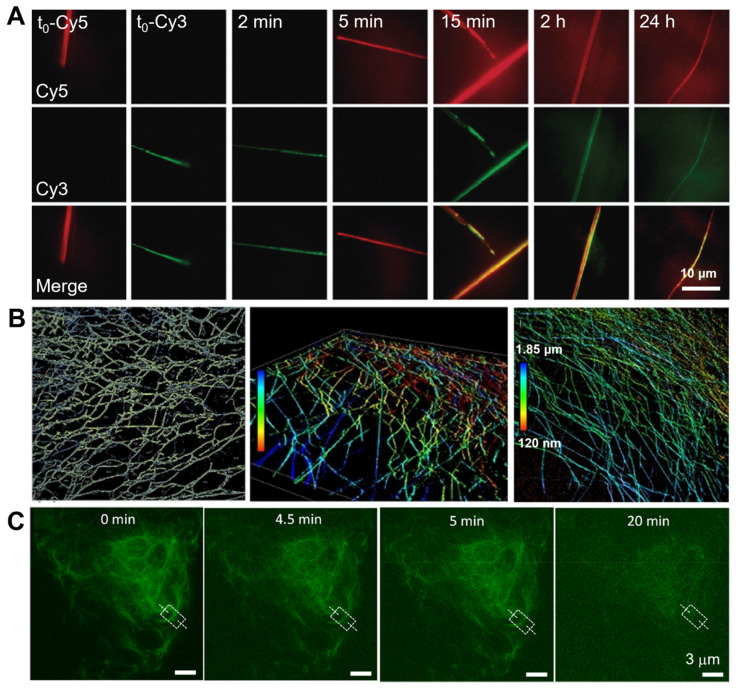
(**A**) Fluorescent images of FF assemblies illustrating the assembly dynamics at different time points. Reproduced with permission from [86]. (**B**) 3D imaging of Fmoc-FF hydrogels and mesh size identification using the PAINT method. Reproduced with permission from [87]. (**C**) In situ and real-time STED imaging demonstrating the disintegration of peptide-based supramolecular nanofibers over time. Reproduced with permission from [88].

Using PAINT imaging with a high spatiotemporal resolution, Albertazzi and co-authors discovered the dynamic and structural features of diphenylalanine (FF) nanofibers schematically [86]. Through the multicolor SRM imaging and two-color kinetic experiments, the FF nanofibers were found to be dynamic, exchanging monomers over time until reaching a thermodynamic equilibrium, which is indicated by the Cy5 and Cy3 probes incorporated equally (Figure 3A). It was confirmed that the obtained FF nanofibers exist the heterogenous behavior. Meanwhile, non-exchanged static aggregates also co-exist alongside those that are fully exchanged. Recently, Albertazzi and co-workers identified Fmoc-FF hydrogels through PAINT imaging without labelling the gels. This approach has enabled the visualization of fiber networks with an enhanced resolution, reaching down to approximately tens of nanometers (~50 nm) in both two-dimensional and three-dimensional contexts. This has facilitated the determination of crucial parameters such as fiber diameter and mesh size (Figure 3B) [87]. The PAINT technique opens up avenues for employing super-resolution imaging in the analysis of gels, enabling a direct visualization of the network structure. It provides insights into the spatial variability of important features, aiding in a comprehensive comprehension of hydrogel networks, their assembly processes, and the influence of these factors on their mechanical characteristics.

Moreover, super-resolution fluorescent imaging has also been used to investigate cationic peptide nanostructures. Ulijn et al. presented a general strategy for using electrostatic interaction between the cationic groups of lysine (or arginine) residues exposed on the peptide nanostructure surface and anionic sulfonate groups in Alexa-488 dye for a range of cationic peptide nanofibers super-resolution imaging (Figure 3C) [88]. Through the application of STED, the static peptide nanostructures were visualized with a resolution down to 52 nm, especially the nanostructures that were sufficiently positively charged (zeta potential > 10 mV). The dynamic degradation process of peptide nanofibers using enzymatic disintegration was also directly visualized in situ and in real time, offering a mechanistic understanding of the degradation kinetics as well as presenting crucial information on the previous work on the enzymatic assembly and disassembly of peptides. This study functioned as a prototype and proposed a general approach for the super-resolution imaging of dynamic soft nanostructures within their native aqueous environment, which can be applied to explore bio-inspired active assembly processes.

#### 3.1.3. Protein-Based Molecular Assemblies

**Figure 4:**
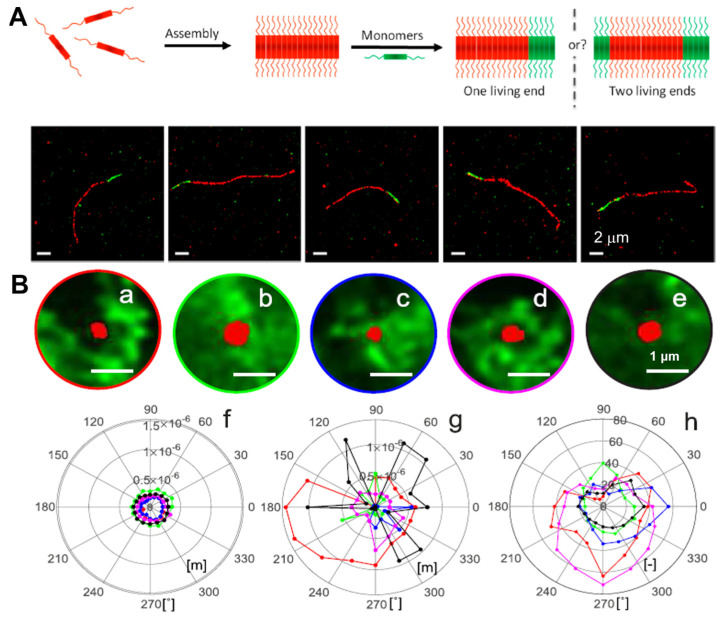
(**A**) The red-green fibrils with a deblock structure investigated using representative STORM imaging revealed the unidirectional living growth of self-assembled protein nanofibrils. Reproduced with permission from [90]. (**B**) Angle-dependent heterogeneity of the distance to the protein maxima and mean protein intensity around a specific fat droplet. Reproduced with permission from [93].

SRM has also demonstrated its efficacy as a potent tool in the realm of protein-based molecular assemblies as well as its involvement with proteins. Albertazzi et al. reported the first non-natural polypeptide that was capable of unidirectional and irreversible fibrillar self-assembly [90]. They elucidated the growth dynamics, exchange kinetics, and polymerization mechanism for fibrils composed of a recombinant triblock protein polymer by employing a combination of AFM and STORM techniques. The reported protein forms fibrils via a nucleation and growth process. Utilizing two-color STORM microscopy, it was found that these protein fibrils are irreversible due to their inability to exchange protein monomers. The obtained fibrils grow unidirectionally, like a “living” polymerization, despite the fairly symmetrical nature of the protein (Figure 4A). This interesting finding paves the way for the designation of multistep hierarchical self-assembly processes. For example, to regulate layered assembly, the manipulation of the sequence when integrating monomers with diverse functions could pave the way for influencing the structures of fibrillar networks.

SRM has also been used to advance the understanding of food materials that are involved with proteins [91,92,93]. Nanoscopic investigation is crucial since certain characteristics are influenced by the interactions between proteins, carbohydrates, lipids, and colloidal suspensions, which can impact their performance within the intricate environment of food. Anni Bygvrå Hougaard et al. investigated the microstructures of acidified milk gels by adding various whey proteins, through STED microscopy coupled with quantitative image analysis and rheological studies [92]. Their research revealed that both whey protein concentrates and nano-particulated whey proteins had the capacity to self-assemble and bind to casein aggregates via intermolecular cross-linking. All the nano-particulated whey protein, liquid casein, and whey protein concentrates behave in a similar way to each other, with the exception that the nano-particulated whey protein system formed larger aggregates and demonstrated an enhanced connectivity with the gel network. In contrast, micro-particulated whey protein did not engage in interactions with other proteins, and its dispersed particles were discernible within the composite gels. The degree of spatial colocalization of fluorescence emissions from casein and whey protein was most pronounced in the system that solely comprised endogenous proteins. This method assists the industry in refining the application of various ingredients and in creating novel, adequately stable fermented dairy products.

As with egg white proteins, it was proved that materials with very different textural attributes would generate under different cooking temperatures [91]. STED imaging facilitated a more accurate quantification of the distinct structures, revealing a 7–13 fold increase in the number of particles within the solid phase of the materials [91]. The findings indicated that STED imaging could quantify the enhanced particle count and density, which may be linked to the logarithmic rise in the elastic modulus and the increased fragility of egg white as it was cooked at the three different temperatures. The combination of STED microscopy with macroscopic rheological measurements assisted in gaining the extra information on the understanding of protein aggregation in food matrices. Scientists has also established and applied a 2D spatial cross-correlation analysis methodology to investigate the relative spatial arrangements of protein and fat in acid-induced whole milk gels, using two-channel images that were obtained from STED imaging and confocal microscopy (Figure 4B) [93]. They characterized several parameters, including the protein microstructure, the fat droplet size, and the distances between the protein networks and fat droplets. Significantly different distances between the fat droplets and the protein network in the homogenized samples compared with the non-homogenized sample were also demonstrated. All these reports of the potential applications of SRM in food matrices help to fill a current gap in food science research. In the near future, the development of new functional products and many improvements in food quality are expected to come from the deeper understandings and interventions at the microstructural level. More importantly, all these research objects might strive for further improvement with the assistance of SRM.

#### 3.1.4. DNA-Based Molecular Assemblies

DNA nanostructures are acknowledged as one of the most promising candidates to serve as nanocarriers in drug delivery systems, owing to their structural programmability, flexibility, and biocompatibility [94,95,96]. Initially developed by Rothemund et al. in 2006, DNA origami can be formed by folding long, single-stranded viral genomic DNA (scaffold strands) into arbitrary shapes with the assistance of numerous short oligonucleotides (staple strands) [97]. With the programmable assembly of nanoscale molecular structures, the field of DNA nanotechnology has been revolutionized by the DNA origami technique, which allows molecular engineers to construct 2D and 3D structures with almost any arbitrary shape [36,97,98,99]. The small size of DNA nanostructures had previously precluded the application of fluorescence microscopic methods due to the constraints imposed by the diffraction limit. However, the application of fast and powerful SRM in recent years has facilitated the rapid development of DNA nanotechnology [100].

Multiple types of SRM equipment have contributed to the structure investigations of DNA-based materials, revolutionizing the way that we comprehend the DNA family, including blink microscopy (BM), binding-activated localization microscopy (BALM), dSTORM, single-molecule high-resolution imaging with photobleaching (SHRImP), quantitative PAINT (qPAINT), DNA-PAINT, exchange PAINT, proximity-dependent PAINT (PD-PAINT), and Förster resonance energy transfer PAINT (FRET-PAINT) [54,101,102,103,104]. The critical advances in SRM techniques have helped to demonstrate the organization, damage, and repair of DNA organization and enabled unprecedented insights into DNA structures, functions, and many other new discoveries [105]. The combination of a molecular breadboard DNA origami and an analytical SRM tool will assist in the construction of new kinds of bottom-up nanoscale structures, as well as in making the detection of quantitative information and molecular interactions come true [102].

**Figure 5:**
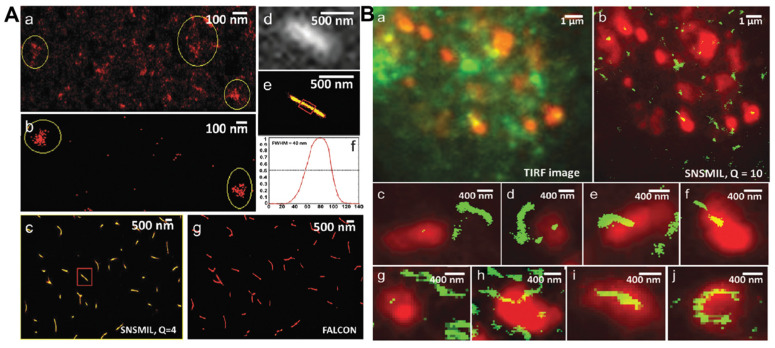
(**A**) BALM and reconstructed images of triangle DNA origami nanostructures. (**B**) Different position relations and interactions between the tube DNA origami and lysosomes in NIH 3T3 cells. Reproduced with permission from [106].

BALM is based on the principle that the fluorescent dye YOYO-1 could show a strong fluorescence enhancement upon binding to double-stranded DNA [38,106]. Li and co-authors used BALM to image triangle DNA origami for the first time with a dramatic resolution enhancement [106]. The intricate geometry and small size of triangle DNA origami also present stringent demands on the localization precision and algorithms, and both FALCON and SNSMIL have demonstrated the capability to visualize tubular structures (Figure 5A). The synthetic DNA nanostructures in NIH 3T3 cells, also illustrated using BALM imaging, revealed the detailed interactions through the combination of the TIRF images of lysosomes and the fluorescence localization images of the tube DNA origami nanostructures (Figure 5B) [106]. The capture as well as the degradation by lysosomes with the time of the tube DNA origami have also been illustrated. Furthermore, the chromosomal organization in fixed Escherichia coli cells was imaged using BALM, through a strong fluorescence enhancement emitted by YOYO-1 as it binds to double-stranded DNA [38]. The precise colocalization of proteins with DNA was achieved through BALM enhanced by YOYO-1 and PALM assisted by genetically encoded fluorescent protein tags, yielding a resolution down to a few base pairs.

**Figure 6:**
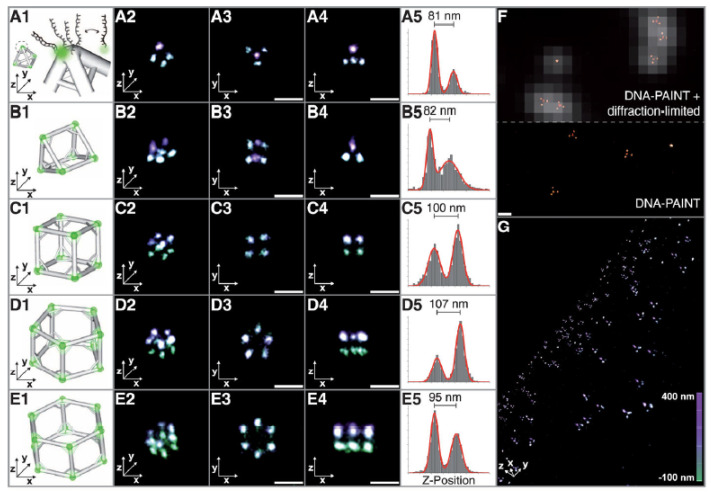
3D DNA-PAINT super-resolution fluorescence imaging of a polyhedral. Reproduced with permission from [107].

Utilizing DNA-PAINT, Simmel and colleagues explored the dynamic behavior of DNA nanostructures, enabling the routine analysis of DNA binding and unbinding kinetics. This approach further allowed for the determination of the varied parameters of the binding sites on the nanostructures and the corresponding kinetic rates [39]. They have demonstrated ribbon-like, flat, monomeric DNA structures as well as multimeric DNA structures by using the reversible specific binding of labeled oligonucleotides to DNA nanostructures in PAINT imaging. Lately, using DNA-PAINT in solution, Jungmann and co-workers accurately constructed and characterized the 3D polyhedra structures, including tetrahedron, triangular, pentagonal, and hexagonal prisms, and cubes from DNA tripods. They achieved an impressive resolution of roughly 13 nanometers along the x and y axes, and about 24 nanometers along the z axis (Figure 6) [107]. As 3D analogs to the 2D rectangle, DNA origami barrels were also presented [108]. The modularity of DNA barrel structures has facilitated the swift adoption of this technology across a wide range of fields, extending its reach beyond the specialist communities engaged in DNA nanotechnology [108]. SMLM encoded the peptide–protein interaction upon cellular fixation with a novel peptide–PAINT probe without additional labeling [109] and a DNA origami platform in super-resolution could also quantify the protein copy number [110].

Moreover, Yin and co-workers further demonstrated an Action-PAINT strategy for super-resolution labelling upon visualization on single molecules [111]. The reported approach monitored and localized DNA binding events in real time with DNA-PAINT, and upon the visualization of binding to a desired location, photo-crosslinks the DNA to affix the molecular label. This method opens up a broad range of new biological investigations and demonstrates a high (59–65%) on-target labelling efficiency on a synthetic DNA nanostructure breadboard, which enables targeted single-molecule labelling upon visualization at the single-molecule scale, allowing the biological researchers to not only ‘see the previously invisible’ but to also ‘touch the previously inaccessible’ [111]. Despite the single SRM research method, the correlative microscopy has further enhanced the characterization of DNA nanostructures by offering a high specificity, fidelity, and resolution, as well as enabling quantitative imaging. Examples of such advanced techniques include spinning disk confocal microscopy (SDC)/DNA-PAINT, DNA-PAINT/AFM, single-molecule FRET/DNA-PAINT, and STORM/expansion microscopy (ExM) [63,112,113,114].

### 3.2. Organic–Inorganic Hybrid Assemblies

Organic–inorganic hybrid materials with complicated structures and components have fulfilled various biological functions, such as defense, protection, and mechanical support in living organisms [115,116]. As the principal inorganic constituents of biological hard tissues (teeth and bone) [117], calcium phosphate (CaP) and calcium carbonate (CaCO_3_) are regarded as the most widely researched biomineralization organic–inorganic hybrid materials [118,119,120]. In recent years, SRM has been widely used to study biomineralization, which might present more dynamic process information and organic component distribution, helping to understand the whole dynamic process of crystal nucleation, crystal growth, and phase transformation [121,122,123,124,125,126,127]. Combined with fluorescent labeling or functionalized quantum dots (QDs) as fluorescent labels, SRM can be used as a powerful technique to detect the distribution of numerous components and their interactions between different organic constituents, encompassing proteins and key constituents or organic materials within organic–inorganic hybrid systems of diverse shapes. This approach promises to offer novel insights into the mechanisms of biomineralization.

**Figure 7:**
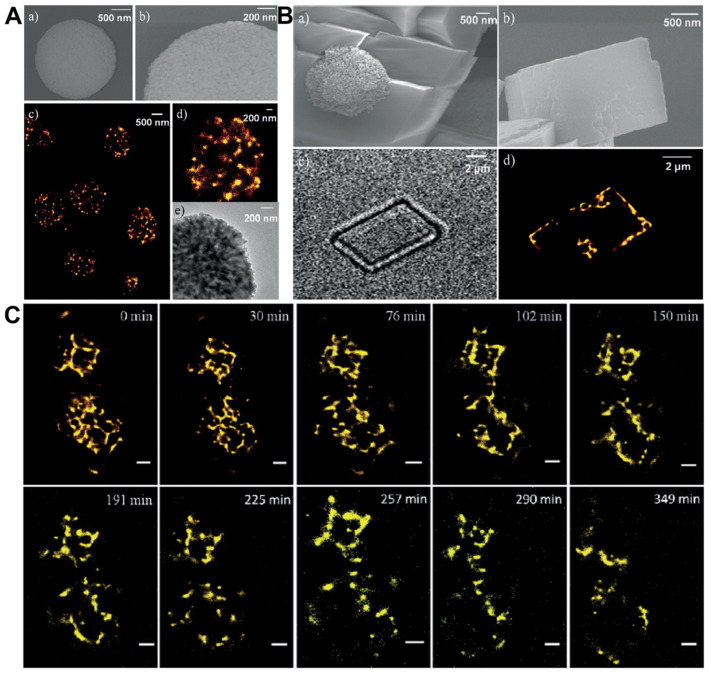
(**A**) Nanostructures of the aimed vaterite microspheres and the gelatin distribution. (**B**) Structures of calcite rhombohedra and the distribution of gelatin. (**C**) The distribution of gelatin in CaCO_3_ over time investigated using dSTORM imaging in a switch buffer, Scale bar: 1 μm. Reproduced with permission from [121].

dSTORM/STORM/PALM was introduced to detect the biomineralization of CaCO_3_, providing a direct observation of the dynamic interaction between the inorganic and organic phases, further expanding the understanding of the important regulatory role of biomacromolecules in the process of biomineralization [121,122,123,124]. The dSTORM images showed that gelatin is distributed in vaterite microspheres with a form of nanoparticles, and gelatin tended to accumulate on the edge of calcite rhombohedra, which is different from the previous report that the silk fibroin (SF) distributed in vaterite by a homogeneous formation verified by elements mapping (Figure 7A) [121]. The dSTORM image also shows that gelatin tended to accumulate on the edge of calcite rhombohedra (Figure 7B). Furthermore, the time-dependent process of gelatin excluded from the CaCO_3_ crystals during the transformation was also monitored (Figure 7C). The distribution patterns of the nacre proteins inside the whole synthetic calcite with different forms were also directly visualized using STORM imaging, which includes islet-like, chain-like, and haze-like protein clusters in the crystals [122]. It is proven that Matrix proteins could promote nucleation during the crystallization process of amorphous calcium carbonate [123] and other research has found that Matrix proteins tend to be preferentially deposited on the rough surfaces of crystals during crystallization, rather than on the smooth and common faces of calcite [124].

The endogenous organic molecule dopamine-mediated biomineralization of CaP as a strategy to easily synthesize functionalized hybrids has been proven using dSTORM [125]. The organic component dopamine (DA) is distributed in the form of nanoparticles and the minority is monodispersed, which is quite different from the EDS mapping. The direct observation of dopamine distribution within the hybrids aids in comprehending the physical chemistry mechanism underlying biomineralization. Similarly, most of the α-amylase is proven to be distributed in the form of nanoparticles in the α-amylase/CaP hybrids using dSTORM imaging, which provided more precise information on the protein inside the flowerlike CaP hybrids.

Except for CaCO_3_-/CaP-based hybrid assemblies, the metal–organic framework (MOF)-based hybrids were also explored using single-molecule localization microscopy [128]. Ge et al. obtained protein@metal-organic frameworks (P@MOFs) via the coprecipitation process and employed dSTORM to resolve the specific three-dimensional localization of the protein inside the P@MOF with ZIF-8 as the matrix. Using a combined methodology of SRM and a clustering analysis, they discovered that enzyme molecules form clusters with metal ions and organic ligands and participate in the coprecipitation process, contributing to both the nucleation and subsequent crystal growth. Furthermore, the internal structures of nanocomposite crystals were characterized using 3D STORM, pinpointing the locations of fluorescent nanoparticles within individual calcite crystals. This technique offered an independent confirmation of the development of dislocation loops with distinctive geometries upon the nucleation of calcite at substrates, thereby providing deeper insights into the processes by which additives are incorporated within the crystal lattice [126].

Apart from the mineralized organic–inorganic materials, the hybrids composed of biomolecules and nanoparticles (NPs) have garnered significant interest and have been thoroughly investigated using SRM [129,130,131,132,133]. “Biomolecular corona” can be formed on the NP surface immediately when exposed to biofluids [134,135,136]. The nanomaterial–“biomolecular corona” constitutes a dynamic entity that establishes a synthetic–natural interface, which can mediate the cellular internalization and subcellular trafficking of nanomaterials within biological systems [137,138,139]. Introducing the SRM to the research of the “biomolecular corona” would significantly enhance our understanding of the development of nanomedicine at the molecular level [20,140,141,142,143,144,145,146]. STORM was employed to quantitatively examine the variation in the penetration depth of different proteins within the porous silica nanoparticles [142].

A non-invasive technique for both visualizing and analyzing the protein adsorption into porous materials was established by the authors, achieving the time-resolved investigation of protein adsorption. This approach yields crucial insights into the formation of the protein corona. The direct visualization, coupled with the mathematical analysis of protein penetration into porous materials, offers detailed information about the composition of the protein corona. All the previous reports will accelerate the development of therapies based on the designation of effective particles and the functional protein corona. dSTORM was also employed to investigate and capture various protein coronas that form on MSN nanoparticles with varying surface chemistries. This technique showcased the dynamic behavior and heterogeneity of protein coronas [140].

**Figure 8:**
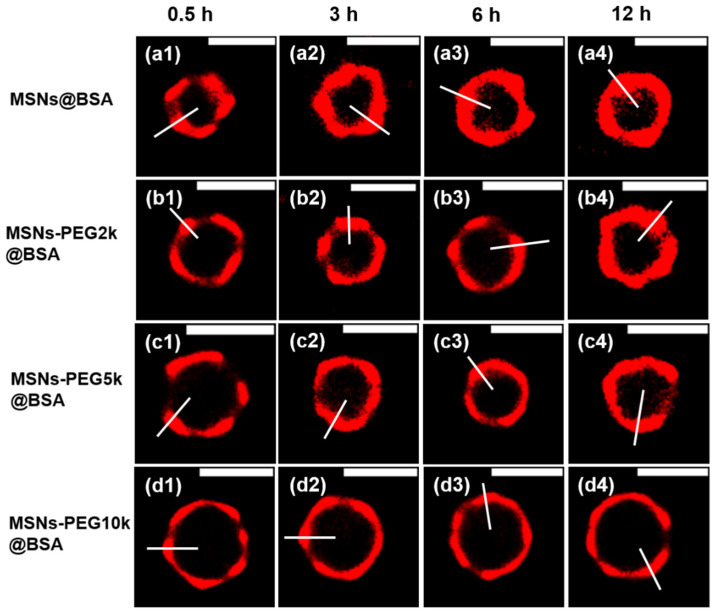
Zoomed dSTORM images of protein adsorption onto MSNs and MSNs-PEGx (x = 2k, 5 k, 10 k) particles after incubation with BSA solution for different timescales (Scale bars: 1 μm). Reproduced with permission from [147].

The use of dSTORM for the direct visualization and quantification of protein coronas on PEGylated mesoporous silica nanoparticles was illustrated (Figure 8) [147]. The authors were devoted to investigating the trends in the protein penetration depth in relation to the incubation duration and the molecular weight of PEG. The findings indicated that the deepest penetration depths present a slight increase with an extended incubation time, whereas they tend to remarkably decrease as the length of the modified PEG chain increases. This report provides insights and a comprehensive understanding of the protein corona formed on PEGylated mesoporous silica particles. Gaining critical insights into nano-biomolecule interactions is a significant focus for the advancement of materials in biomedical contexts, which might promote the development of a biomolecular corona engineering application [138,139].

### 3.3. Polymer Assemblies

Synthetic polymers are macromolecules in which small structural units are connected by covalent bonds [148]. Supramolecular polymers are constructed by monomers that are linked by non-covalent bonds instead of covalent bonds, and they typically include hydrophobic interactions and hydrogen bonding [148]. Their modularity and responsiveness to different stimuli, together with their dynamic nature, means they are extensively used in technology and everyday life and are promising candidates for several applications in optoelectronics, catalysis, biomedicine, and sensing [148]. Over the decades, various instruments and methodologies have been applied to elucidate the morphology and architecture of polymers. How to decode the formation mechanisms, as well as the bulk structures of different polymers, have fascinated researchers for a long time. The cutting-edge SRM tools have had rapid and widespread use in biology and related fields for a long time; however, their implementation in materials, and more specifically in polymer science has been very slow [149,150,151]. Nevertheless, several reports have demonstrated that SRM is a powerful method for studying the dynamics and structures of polymers as well as supramolecular polymers, providing abundant complementary information compared with that attained with ensemble techniques (circular dichroism, UV spectroscopy, and X-ray scattering), and conventional techniques without the need for fluorescence label imaging (TEM, SEM, and AFM) [149]. Based on recent reports, SRM has also been served as a powerful technique in polymer science, including structural characterizations and polymerizations, solution and self-assembly behaviors, bulk structures and behaviors, gel structures and behaviors, phase transitions, and crystallizations [149].

**Figure 9:**
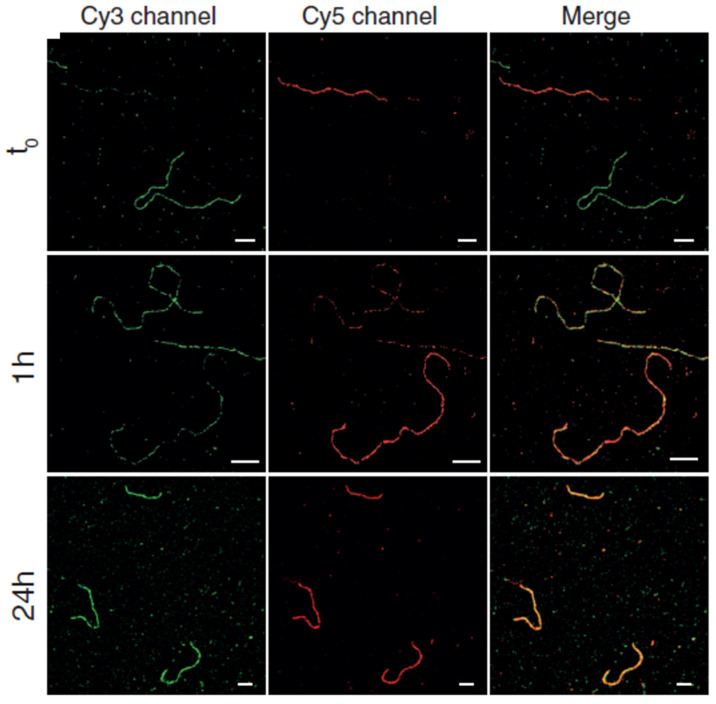
STORM imaging of Cy5- and Cy3-labeled BTA polymers at different mixing time points (Scale bars: 1 μm). Reproduced with permission from [153].

To date, many reports state the sturdiest realization of optical nanoimaging with sub-diffraction resolution for the solution of the self-assembly of block copolymers [149,152,153]. Locating and tracking specific monomers in a mixture of different components can also be investigated using SRM with nanometric resolution and specific labelling. The first example of using SRM in supramolecular polymer imaging was reported by Albertazzi and Meijer, presenting STORM imaging of supramolecular polymers based on the 1,3,5-benzenetricarboxamide (BTA) motif [153]. By preparing different molecules of BTA, the dye-labeled variants of BTA-Cy5 and BTA-Cy3, the authors researched the monomer exchange mechanism of water-soluble BTA supramolecular polymers with the assistance of a particular STORM approach. The two-color STORM method was employed to achieve the dynamic information of the exchange of monomers between fibers, using a static analysis technique. The temporal information can be imprinted into the spectral information, by tagging two sets of assemblies with two different spectrally distinguishable dyes, and by incorporating red-labeled monomers into green-labeled assemblies, and conversely, at defined time intervals (Figure 9). By using a combination of stochastic modelling and STORM, the molecular pathway revealed that an unexpected homogeneous exchange takes place across the entire backbone of the self-assembled BTA fibrillar structures, which was different from the previous hypothesis suggesting that monomer exchange along the fiber occurs only at its termini [153]. The block copolymer micelles, which were assembled from polystyrene-block-poly (ethylene oxide) block copolymers (PSt-b-PEO), were visualized by Zhu and co-workers, through the optical nanoimaging of SRM imaging by staining the polystyrene blocks with spiropyrans (SPs) [152]. The reversible fluorescence on–off switching at an alternating irradiation of UV and visible light could be obtained using SP molecules localized in the hydrophobic phase of the block copolymer micelles, enabling the optical nanoimaging of the microphase structures of the block copolymer self-assembly at a 50-nm resolution.

Lately, similar studies by Meijer et al. performed using the STORM techniques have unveiled the monomer exchange rates of multicomponent supramolecular polymers as well as the influence of chirality on the dynamics of a water-soluble supramolecular polymer in a water environment [154,155]. Using a combination of STORM with other techniques, including FRET, small-angle X-ray scattering (SAXS), and molecular dynamics (MD) simulations, the difference between the fibers and the behavior of the achiral and chiral polymer assemblies on multiple length and timescales were investigated [155]. Molecular changes in the monomers did not greatly disturb the supramolecular structure [155]. Combining STORM with FRET techniques, the formation and dynamic behavior of a bioactive multicomponent supramolecular polymer were investigated [154]. With the created peptide–dye–monomer conjugate, the degrees of monomer incorporation could be measured. Further research demonstrated the equal distribution of monomers within the supramolecular polymer instead of heterogeneous distribution. By tracking the movement of the monomers, researchers also uncovered the diminutive differences in the dynamics of the bioactive monomers.

Moreover, methods including iPAINT, PALM, and STED were reported to enable the super-resolution imaging of supramolecular structures in organic media [156,157,158]. Voets et al. introduced a synthesis-free method that enabled the visualization of dynamic supramolecular architectures in non-polar organic media, by adapting iPAINT microscopy [157]. The quasi-permanent labeling of the fibers is pivotal for achieving an exceptional resolution in the depiction of supramolecular microarchitectures, which was established through a comprehensive series of control experiments. Two-color iPAINT experiments demonstrated the versatility of this approach, unveiling nanometer-thin, micrometer-long supramolecular block copolymers. Manners and co-workers demonstrated the use of SMLM and STED to visualize the self-assembly processes of living crystallization-driven block copolymers (BCPs) in organic solvents at the sub-diffraction scale [158]. The reduction in FWHM from 383 to 76 nm demonstrated the resolution improvement from wide-field microscopy to SMLM. Single-color super-resolution imaging determined the micelle length distributions and BCP nanostructures in situ, with the assistant of four different dyes. Dual-color SMLM was performed on triblock co-micelles to investigate the micelle growth at both seed termini. Moreover, the addition rate of red fluorescent BCP to the termini of green fluorescent seed micelles was measured and compared using dual-color SMLM imaging, which was found to generate block co-micelles. All these results highlighted the potential of SRM tools for probing self-assembly dynamics in organic media. Voets and co-workers extended PALM imaging to capture highly dynamic synthetic nanostructures in organic solvents. They successfully visualized the morphology of dynamic, 1D supramolecular polymers formed by hydrogen-bonded small molecules—these are some of the most difficult molecular systems to image and are elusive to other imaging techniques [156]. Methods for the SRM imaging of the supramolecular structures of BTA fibers in methyl cyclohexane in organic media have been implemented and developed. All the mentioned reports demonstrated that in situ visualization using SRM of the structures and the exchange dynamics of such supramolecular polymers in organic media have shed great light on their structure−function relationships and complex polymerization pathways.

## 4. Conclusions and Outlook

In conclusion, in this review, we have elaborated on why SRM can be used in molecular assemblies and highlighted the advancements in the representative application of SRM in the study of molecular assembly. The developments and breakthroughs of SRM imaging modalities have made it possible to unveil molecular-level insights into the sizes, structures, morphologies, components, component distributions, functions, and dynamics of molecular assemblies in their native state for the rational design and optimization of materials, as well as to evaluate the physicochemical properties of molecular assemblies. SRM imaging realizes the visualization of not only the static architectures of molecular assemblies at the sub-nanometer level, but also their dynamic behaviors over a wide range of time scales (milliseconds to hours). This capability paves the way for insights into the co-assembly and hierarchical self-assembly processes of functional materials.

Despite the mentioned advantages and improvements of SRM and its contribution in the in-depth research of molecular assemblies, no technique alone has the miraculous ability to realize all the temporal and spatial scales necessary for the characterization of complex molecular assembly functional materials. Therefore, progress in correlative microscopy will be a representation of a bright perspective. Combining the advantages of distinct imaging modalities, correlative microscopy has emerged as a promising approach for deeper research, allowing the researchers to provide more detailed and comprehensive structural information on the specimen using two distinct microscopic techniques on a relatively large scale. Such cutting-edge microscopic imaging technologies possess the ability of opening up new opportunities for further development and a deeper understanding of synthetic molecular assemblies as well as natural ones, which pioneers a new way for the rational design of novel molecular assembled functional materials.

SRM has made a great contribution in the research of molecular assemblies; however, there are still some limitations that hinder its deeper understanding. For instance, the approaches to address the limitations, from sample preparation protocols to informative outputs, are supposed improved in detail. Multidisciplinary collaboration might be the best solution for these challenges. Some of these challenges are easy to accomplish by chemists, involving fluorescent dye synthesis, fluorescent dye selection, sample preparation, and sample labeling. However, many of the remaining challenges necessitate comprehensive collaboration across various scientific disciplines, such as mechanical engineering, computer science, software programming, physics, and optics and devices. Moreover, multi-disciplinary expert scientists along with experts in microscope manufacturing have to make a mutual effort to provide practical training on the diverse methods of SRM and its vast experimental potential to emerging researchers. This effort is aimed at making this cutting-edge and sophisticated technique more accessible and routine in use. Nonetheless, significant challenges persist, and the field continues to present abundant opportunities for interdisciplinary scientific exploration.

Except for the discussed representative assembling systems, the self-assembly of a relatively new class of nanometric and/or micron-sized building blocks (for instance Janus or patchy-particles), is also a prospective breakthrough point, which will open new doors for the materials with two-sided properties like physical property, chemical property, structures and functions [159]. Although several reports related to Janus materials have been investigated by SRM, there still lots of margin needs to be excavated [160]. Scientist are supposed to devote more efforts to the exploration of Janus materials by using of SRM.

All in all, these rapid developments in microscopic imaging technologies will open up new opportunities for the further development of molecular assemblies, ultimately leading to the rational design of innovative supramolecular assembled materials. The progress both in molecular assemblies and microscopic imaging technologies are the course of mutual promotion. The development of assembled materials science, including the characterization of dynamic assembled structures or functions at the nanoscale, puts forward higher requirements for imaging instruments. The huge progress in the imaging method, devices, and equipment to realize specific imaging tasks at a super-resolution scale has also accelerated the creation of molecular assembly functional architectures with increasing levels of integration and complexity.

## Figures and Tables

**Table 1:** Different super-resolution fluorescence imaging methods.

**Evanescent wave detection**		Near-field scanning optical microscopy (NSOM) [32]
		Hyperlens for sub-diffraction-limited imaging [33]
**High-resolution far-field optical microscopy**	Enhanced resolution far-field optical microscopy	4Pi-confocal fluoresence microscopy [41]
		Structured illumination microscopy (SIM) [42]
		(IM)-M-5 microscopy [43]
	Reversible saturable optically linear fluorescence transition (RESOLFT)	Stimulated emission depletion microscopy (STED) [44]
		Ground-state-depletion fluorescence microscopy (GSD) [45]
		Saturated structured illumination microscopy (SSIM) [46]
		Saturated pattern excitation microscopy (SPEM) [47]
	Single-molecule localization microscopy (SMLM)	Spectral precision distance microscopy (SPDM) [48]
		Ground-state-depletion microscopy followed by individual molecule return (GSDIM) [49]
		Photoactivation localization microscopy (PALM) [37]
		Fluorescence photoactivation localization microscopy (fPALM) [50]
		Stochastic optical reconstruction microscopy (STORM) [51]
		Direct stochastic optical reconstruction microscopy (dSTORM) [52]
		Point accumulation for imaging in nanoscale topography (PAINT) [53]
		DNA-PAINT [39]
		Blink microscopy (BM) [54]
		Binding-activated localization microscopy (BALM) [38]
		Nanometer-localized multiple single-molecule (NALMS) [55]
		Subtracting patterns in defocused imaging to enhance the resolution (SPIDER) [56]
		Integrating exchangeable single-molecule localization (IRIS) [57]
